# Metabolic fingerprinting of *Angelica sinensis* during growth using UPLC-TOFMS and chemometrics data analysis

**DOI:** 10.1186/1752-153X-7-42

**Published:** 2013-03-01

**Authors:** Yiyun Qian, Yali Wang, Rina Sa, Hui Yan, Xinbo Pan, Yingwen Yang, Yujing Sun

**Affiliations:** 1 Key Laboratory of Dunhuang Medicine and Transformation, Ministry of Education, Research Institute of Angelica sinensis, Gansu University of Traditional Chinese Medicine, Lanzhou 730000, China; 2 Jiangsu Key Laboratory for High Technology of TCM Formulae Research, Nanjing University of Chinese Medicine, Nanjing, China

**Keywords:** *Angelica sinensis*, UPLC-QTOFMS, Metabolomics, Principal component analysis (PCA), Chemometrics

## Abstract

**Background:**

The radix of *Angelica sinensis* is widely used as a medicinal herbal and metabolomics research of this plant during growth is necessary.

**Results:**

Principal component analysis of the UPLC-QTOFMS data showed that these 27 samples could be separated into 4 different groups. The chemical markers accounting for these separations were identified from the PCA loadings plot. These markers were further verified by accurate mass tandem mass and retention times of available reference standards. The study has shown that accumulation of secondary metabolites of *Angelica sinensis* is closely related to the growth periods.

**Conclusions:**

The UPLC-QTOFMS based metabolomics approach has great potential for analysis of the alterations of secondary metabolites of *Angelica sinensis* during growth.

## Background

The root of *Angelica sinensis* (Oliv.) Diels (Umbelliferae), known as Danggui in China, is one of the most important traditional Chinese medicines (TCMs) [1]. It is known as tonic, hemopoetic, spasmolytic, analgesic and anti-inflammatary activities. It is used to treat menstrual disorders, amenorrhea, dysmenorrheal, anemia, constipation, rheumatic arthralgia, traumatic injuries, carbuncles, boils and sores [2]. So far, over 70 compounds have been separated and identified from Danggui, including those from essential oils (mainly including monomeric phthalides), phthalide dimers, coumarins, organic acids and their esters, polysaccharides, polyacetylenes, vitamins, amino acids, and others [3].

Herb is a very complicated system, comprising a complex mixture of different phytochemicals which usually contribute to the therapeutic effect of herbal medicines. Therefore, it is necessary to reflect and control the quality of herbal medicines by a highly effective and comprehensive analysis.

Metabolomics research has emerged as a valuable technology for the comprehensive profiling and comparison of metabolites which are the end products of cellular regulatory processes, and their levels can be regarded as the ultimate response of biological systems to genetic or environmental changes [4]. Metabolomics research comprehensively analyses TCMs or herbal remedies so as to assess their quality, especially when it couples with chemometrics data analysis approach [5-10].

Principal component analysis (PCA) is the most widely used unsupervised chemometrics method in metabolomics studies. Plants that are far apart in the photograph will be very distinct, while plants closer to each other will be more similar [11]. PCA is not only used for classification, such as identifying geographic origin or taxonomic discrimination [12,13], but also used as an approach to analyze the alterations of secondary metabolites of herb medicine during growth so as to get the best harvest time [14,15].

Those above applications of metabolomics research are based on the utilization of large-scale analyzed data of metabolites. Over the past decade, many methods for the high-throughput plant metabolomics analysis have been established and mass spectrometry-based platforms have been most widely used in this field [16]. Among the various MS-based platforms, liquid chromatography coupled with MS (LC-MS) is considered to be particularly important in plants research, since it covers many semi-polar compounds, such as key secondary metabolite groups, which can best be separated and detected by LC-MS approaches [17].

Time-of-flight mass spectrometry (TOFMS) is widely used in metabolomics studies to obtain more accurate and precise MS data [18,19]. TOFMS provides good sensitivity and resolution to profile intact precursor ions which are generated from metabolites through ESI and represents the most appropriate MS instrument to apply LC separations for this objective [16,20].

In this paper, we describe a high-throughput and reliable ultra-performance liquid chromatography/time-of-flight mass spectrometry (UPLC-TOFMS) based analytical method coupled with PCA to profile samples of *Angelica sinensis* during growth. Accurate mass, tandem mass, MassFragment software and UV spectrum were used to identify these significant compounds. The study has shown that accumulation of secondary metabolites of *Anglica sinensis* is closely related to the growth periods.

## Results and Discussion

### UPLC-PDA-MS chromatographic fingerprints of *Angelica sinensis*

**Figure 1:**
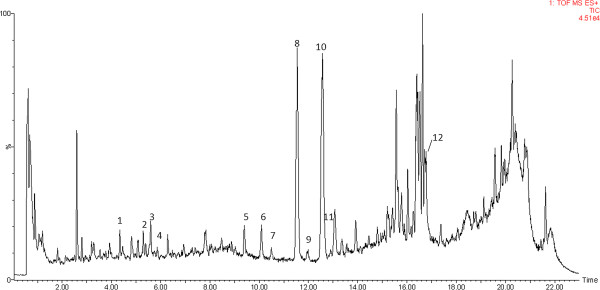
**UPLC-TOFMS ESI**^**+ **^**chromatogram of sample No.14.**

**Figure 2:**
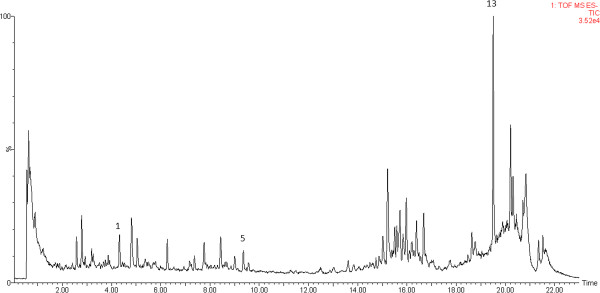
**UPLC-TOFMS ESI**^**- **^**chromatogram of sample No.14.**

**Figure 3:**
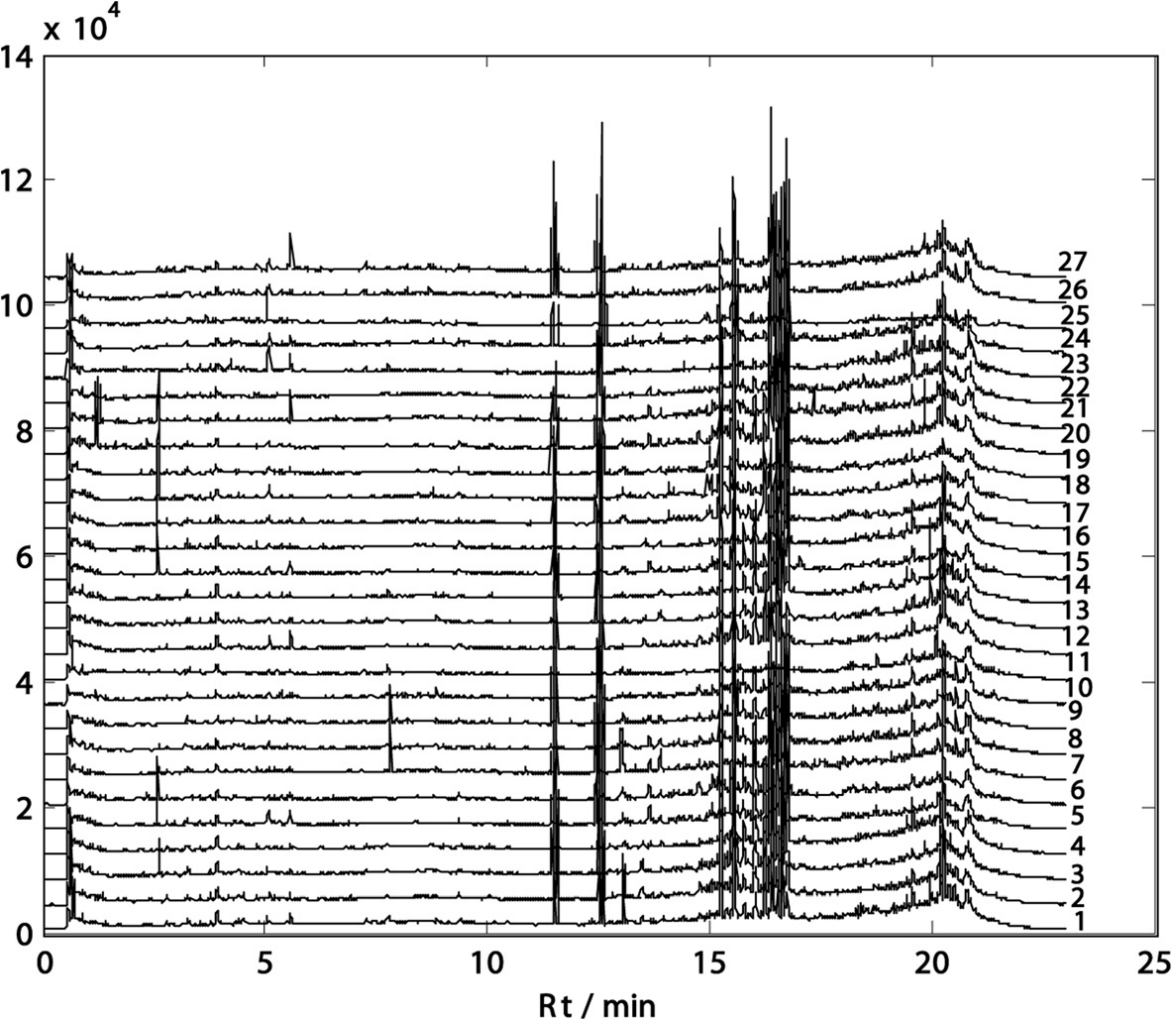
**UPLC-TOFMS ESI**^**+ **^**chromatogram of 27 samples.**

**Figure 4:**
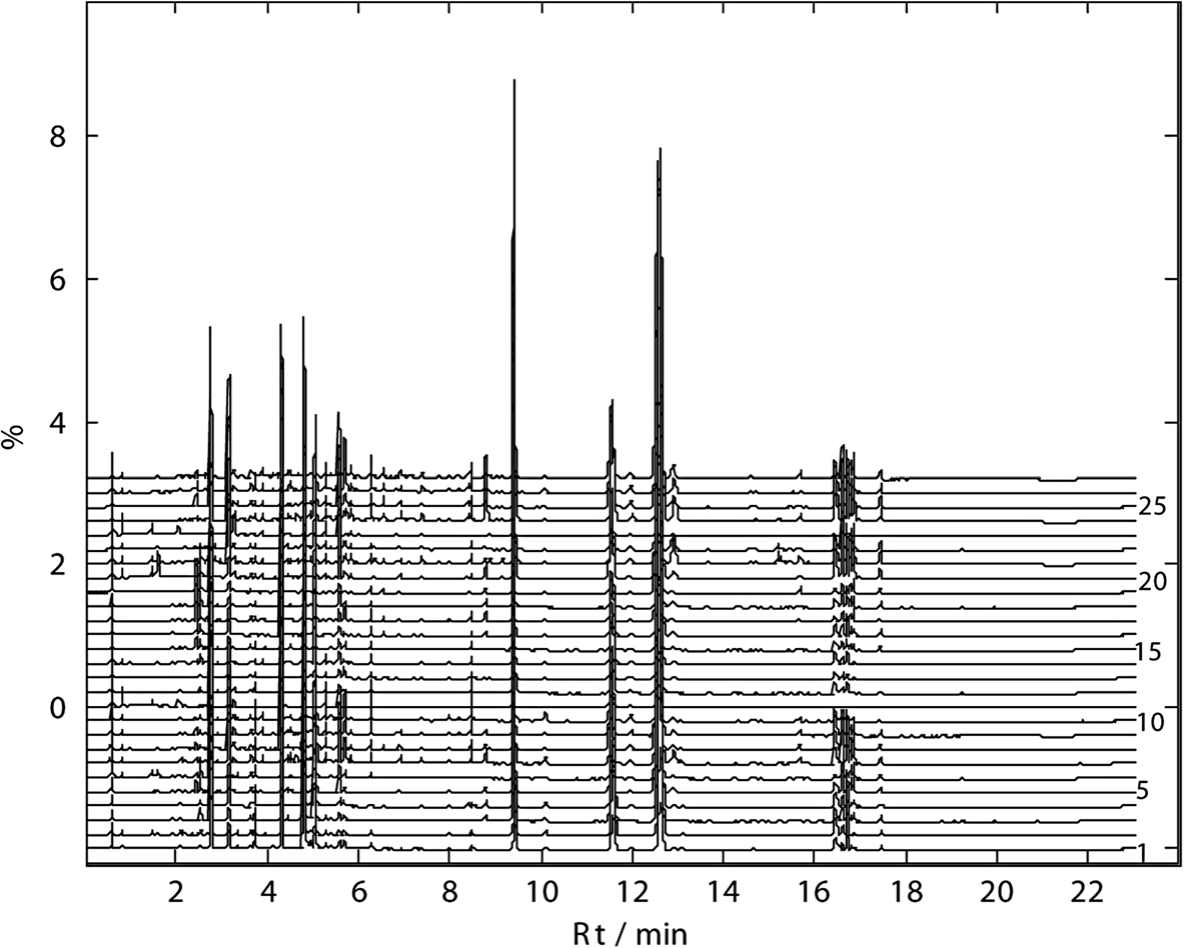
UPLC-UV chromatogram of 27 samples at 280 nm.

UPLC-MS analyses of sample No. 14 in both ESI^+^ and ESI^-^ modes were carried out (Figures 1, and 2). Based on the results, the ESI^+^ mode was finally chosen for the whole analyses since it was more informative than the negative one. The chromatograms of UPLC-TOFMS (ESI^+^) and UPLC-UV (under 280 nm) for all 27 samples were shown in Figures 3, and 4, respectively.

### Identification of components in *Angelica sinensis*

In order to identify components in *Angelica sinensis*, qualitative analysis of compounds in *Angelica sinensis* were carried out on an UPLC coupled to ESI quadrupole time-of-flight mass spectrometry (UPLC-ESI-QTOFMS) in ESI^+^ and ESI^-^ modes. And UV data is also used to provide evidence for identification. The MS and UV data were shown in Table 1.

**Table 1:** **Precursor and product ions and UV spectrum data of compounds in *Anglica sinensis***

**Compound number**	**Identification**	**R**_**t **_**(min)**	**Theoretical accurate mass (m/z)**	**QTOFMS (m/z) (ESI**^**+**^**/ESI**^**-**^**)**	**Mass accuracy (ppm)**	**MS/MS fragment ion (m/z)**	**UV**_**λmax **_**(nm)**
1^a^	Ferulic acid	4.341	195.0657	195.0674	8.7	135.0446[M + H-CH_3_COOH]^+^	239, 322
			[M + H]^+^	[M + H]^+^			
2^b^	E-6,7-Dihydroxydi- hydroligustilide	5.29	207.1021	207.1021	0	207.0946[M + H-H_2_O]^+^	
			[M + H-H_2_O]^+^	[M + H-H_2_O]^+^			
						165.0493[M + H-H_2_O-CH_2_CH_2_CH_3_]^+^	278
						161.0900[M + H-2H_2_O-CO]^+^	
3^b^	Senkyunolide H(I)	5.593	207.1021	207.1000	−2.1	207.0952[M + H-H_2_O]^+^	
			[M + H-H_2_O]^+^	[M + H-H_2_O]^+^			
						189,0849[M + H-2H_2_O]^+^	277
						162.1036[M + H-H_2_O-COOH]^+^	
4^b^	Senkyunolide I(H)	5.855	207.1021	207.1030	4.3	207.0952[M + H-H_2_O]^+^	
			[M + H-H_2_O]^+^	[M + H-H_2_O]^+^			
						189,0849[M + H-2H_2_O]^+^	277
						165.0493[M + H-H_2_O-CH_2_CH_2_CH_3_]^+^	
5^b^	Coniferyl ferulate	9.397	357.1338	357.1298	−4.0	207.0977[M + H-C_8_H_6_O_3_]^+^	
			[M + H]^+^	[M + H]^+^			
						191.1159[M + H-C_8_H_6_O_4_]^+^	269, 317
						162.0766[M + H-C_10_H_11_O_4_]^+^	
6^b^	Senkyunolide A	10.091	193.1229	193.1213	−8.3	193.1284[M + H]^+^	
			[M + H]^+^	[M + H]^+^			
						160.0836[M + H-H_2_O-CH_3_]^+^	280
						147.1206[M + H-H_2_O-CO]^+^	
7^a^	Butylphthalide	10.49	191.1072	191.1093	11.0	191.1055[M + H]^+^	
			[M + H]^+^	[M + H]^+^			
						145.1036[M + H-H_2_O-CO]^+^	257
						117.0733[M + H-COOH-CH_2_CH_3_]^+^	
8^b^	**E-Ligustilide**	11.539	191.1072	191.1068	−2.1	191.1091[M + H]^+^	
			[M + H]^+^	[M + H]^+^			
						173.0979[M + H-H_2_O]^+^	328
						163.1151[M + H-CO]^+^	
9^b^	E-Butylidenephalide	11.989	189.0916	189.0907	−4.8	153.0685[M + H-2H_2_O]^+^	
			[M + H]^+^	[M + H]^+^			
						143.0854[M + H-H_2_O-CO]^+^	261, 310
						115.0587[M + H-COOH-CH_2_CH_3_]^+^	
10^a^	**Z-Ligustilide**	12.569	191.1072	191.1085	6.8	191.1064[M + H]^+^	
			[M + H]^+^	[M + H]^+^			
						173.0967[M + H-H_2_O]^+^	280, 328
						163.1062[M + H-CO]^+^	
11^a^	Z-Butylidenephalide	12.887	189.0916 [M + H]^+^	189.0908 [M + H]^+^	−4.2	153.0727[M + H-2H_2_O]^+^	
						143.0826[M + H-H_2_O-CO]^+^	260, 311
						105.0312[M + H-C_5_H_8_O]^+^	
12^a^	**Levistolide A**	16.776	381.2066	381.2075	2.4	363.2019[M + H-H_2_O]^+^	
			[M + H]^+^	[M + H]^+^			
						345.1783[M + H-2H_2_O]^+^	288
						335.1978[M + H-COOH]^+^	-
13^a^	Linoleic acid	19.501	279.2314	279.2314	−3.6		
			[M-H]^-^	[M-H]^-^		191.1714[M-H-C4H10O2]^-^,	-
						149.0979[M-H-C8H20O]^-^	-

a. and b. refer to the different approaches of identification and significant variable marker ions were bold.

a. Reference standard. b. Q-TOFMS tentatively identified.

The potential calculated masses and elemental compositions associated with the measured mass of the compounds were generated and studied with MassLynx. Among them, six compounds were identified to be ferulic acid(**1**), Z-ligustilide(**7**), butylphthalide(**10**), Z-butylidenephalide(**11**), linoleic acid(**12**), levistolide A(**13**), respectively, by comparing the accurate mass and retention time with those of standards.

The remaining seven compounds were provisionally identified to be E-6,7-dihydroxydihydroligustilide(**2**), senkyunolide H(**3**), and senkyunolide I(**4**) [21,22], coniferyl ferulate(**5**) [21,23], senkyunolide A(**6**) [21,23], E-ligustilide(**8**), and E-butylidenephalide(**9**) [23], respectively, by comparing their MS (accurate and tandem mass) and UV data with reported values. UV spectrum data and retention time were compared with that in literatures. When the reference compound was not available, MassFragment software increased the confidence and facilitation in the MS fragment analysis of the proposed structure.

By examining compounds **2**, **3** and **4**, it was found that their MS all exhibited the protonated molecular ion at m/z 207. Abundant product ions of these three compounds were shown in Table 1. Their MS data and UV spectrum were compared with those in literatures and were consistent with E-6,7-dihydroxydi-hydroligustilide, senkyunolide H and senkyunolide I [21,22]. Therefore, compounds **2**, **3** and **4** were tentatively identified as E-6, 7-dihydroxydi-hydroligustilide, senkyunolide H and senkyunolide I.

The MS of compound **5** showed a protonated molecular ion [M + H]^+^ at m/z 357.1298. The [M + H]^+^ ion of compound **5** gave abundant product ions at m/z 191.1159 (loss of C_8_H_6_O_4_) and m/z 162.0766 (loss of C_10_H_11_O_4_). These characteristic fragment ions were highly compatible with the structure of coniferyl ferulate. According to its MS and UV spectra and published data [21,23], the compound 5 could be tentatively assigned as coniferyl ferulate.

Compound **6** showed a protonated molecular ion [M + H]^+^ at m/z 193.1213 and gave abundant product ions at m/z 160.0836 (loss of H_2_O-CH_3_) and 147.1206 (loss of H_2_O-CO). Moreover, its UV spectra were equivalent to the literatures value for sendanenolide A [21-23].

Compounds **8** and **9** were tentatively assigned as E-ligustilide and E-butylidenephalide by comparing with the MS and UV data of Z-ligustilide and Z-butylidenephalide (reference standards) and published data [23].

**Figure 5:**
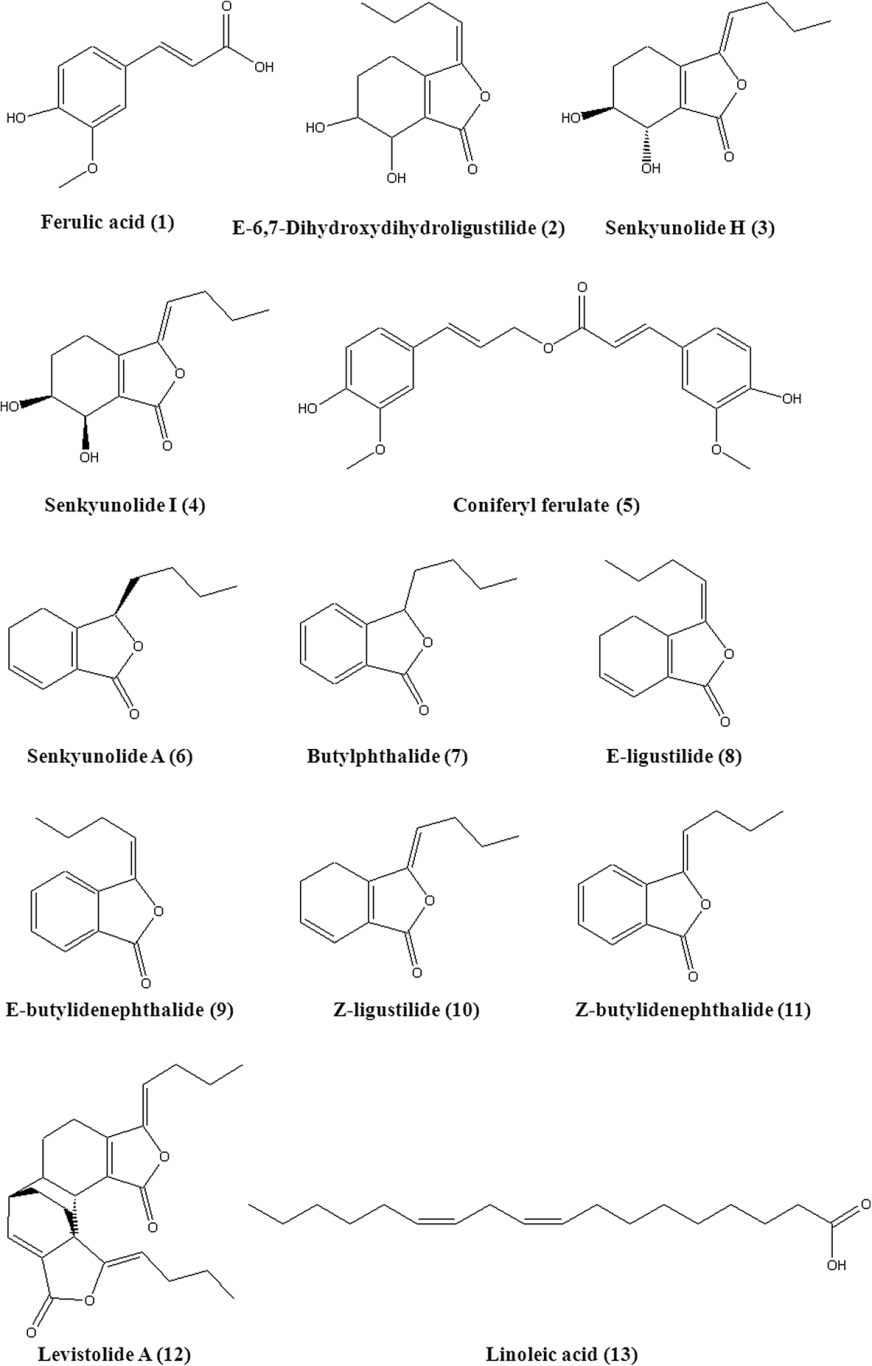
**Chemical structures of the identified compounds in *Angelica sinensis.***

The structures of identified compounds and their related MS, UV data were shown in Figure 5, and Table 1, respectively.

### Principal Component Analysis (PCA)

**Figure 6:**
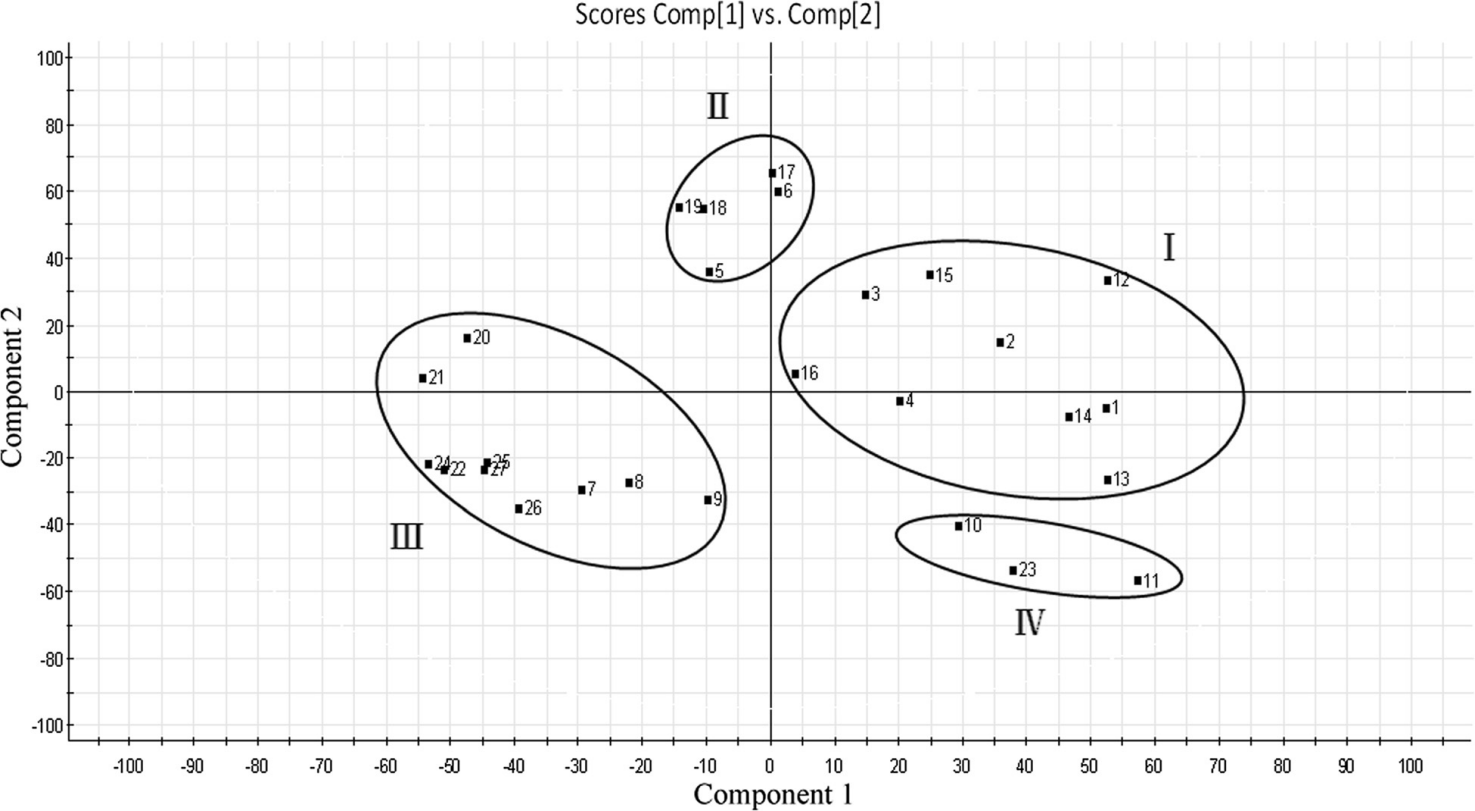
**PCA score plot of *Angelica sinensis *from different growth period.**

For analysis of 27 samples in different growth periods, an unsupervised pattern recognition method, PCA, was performed. PCA was used to visualize the relationship among samples. The clear separation of these 27 samples was observed in the PCA scores plot where each coordinate represents one sample (Figure 6). The PCA scores plot in Figure 6 could be readily divided into four relative groups: I (Sample No.1, 2, 3, 4, 12, 13, 14, 15 and 16), II (Sample No. 5, 6, 17, 18 and 19), III (Sample No. 7, 8, 9, 20, 21, 22, 24, 25, 26 and 27), IV (Sample No. 10, 11, 23) indicating that the content and distribution of compounds were highly varied in these 27 samples.

Samples in group I (Sample No.1, 2, 3, 4, 12, 13, 14, 15 and 16) were collected in first year of growth period and at the end of August to the end of October in second year. Root of *Angelica sinensis* grows and accumulates metabolites very fast during this period. A large number of assimilation products are transported from leaves to root during these months [24]. Group II contains samples which collected in traditional collecting time (Sample No. 17, 18 and 19). It is indicated that sample 5 and 6 which collected in the March and April were have similar content and distribution of compounds with samples which collected in traditional collecting time. Samples in group III were collected from May and June which is the period that aerial parts grow, and other samples in group III (Sample No.24, 25, 26 and 27) were collected after bolting and flowering, which indicated that samples in the period of aerial parts grow fast have similar chemical compounds. Before functional leaf works, root of *Angelica sinensis* works as a nutrient reserve. It supplies organic matter for taking root and sprouting. Organic matter which produced by leaves is depleted during taking root and sprouting before it transported to root [25,26].

### Tentative marker assignment

**Figure 7:**
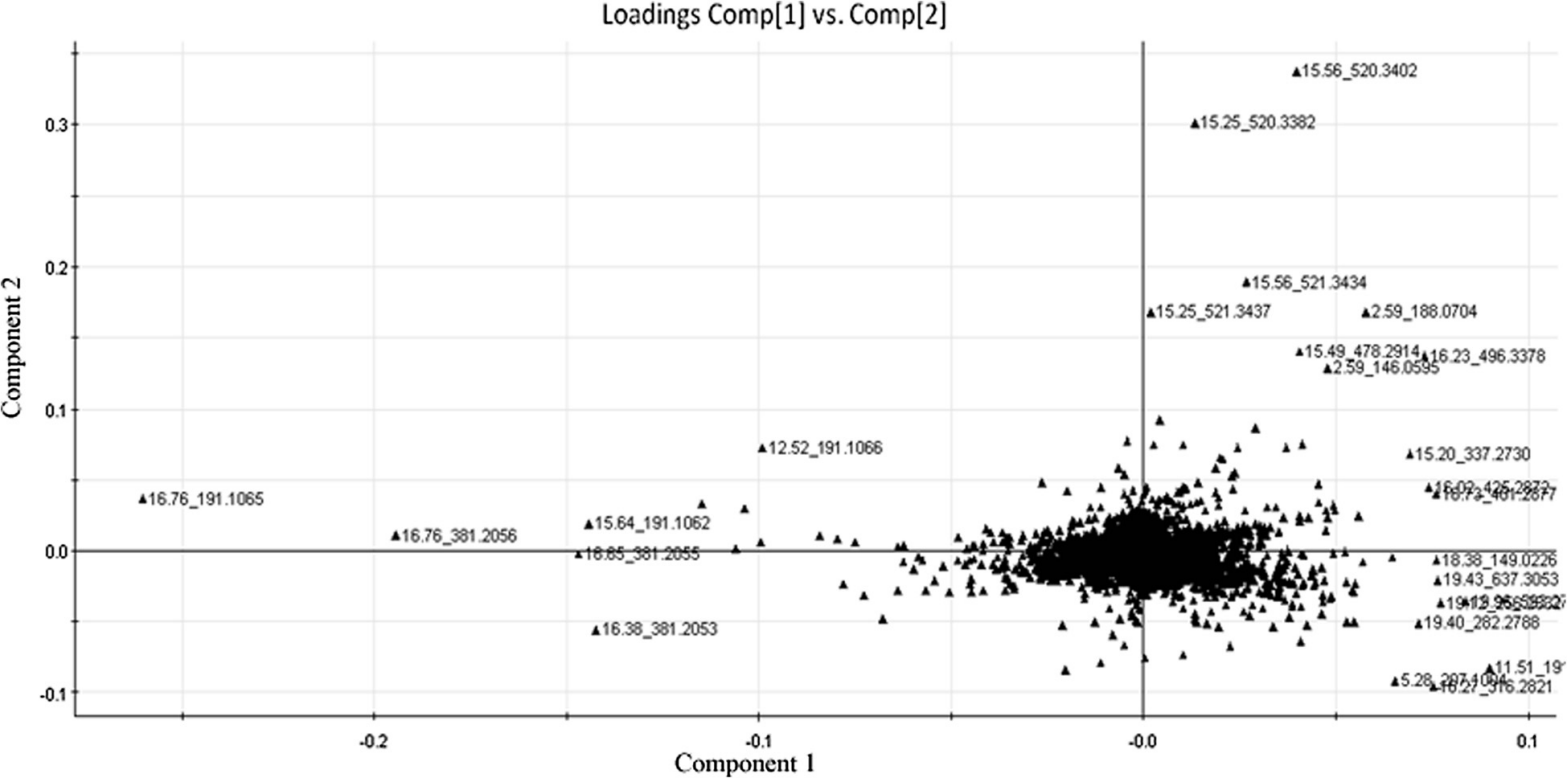
**Loadings plot of *Angelica sinensis *from different growth period.**

It is possible to determine variable importance by analyzing the correlation between variables in the PC1 and PC2 dimension, a list of interest was therefore obtained from the PCA loadings plot (Figure 7). Marker ions m/z 146.0595 ([M + H]^+^, Rt 2.59 min), 188.0704 ([M + H]^+^, Rt 2.59 min), 207.1004 ([M + H]^+^, Rt 5.28 min), 191.1068 ([M + H]^+^, Rt 11.51 min), 191.1066 ([M + H]^+^, Rt 12.52 min), 520.3382 ([M + H]^+^, Rt 15.25 min), 478.2914 ([M + H]^+^, Rt 15.49 min), 520.3402 ([M + H]^+^, Rt 15.56 min), 521.3434 ([M + H]^+^, Rt 15.56 min), 191.1062 ([M + H]^+^, Rt 15.64 min), 496.3378 ([M + H]^+^, Rt 16.23 min), 316.2821 ([M + H]^+^, Rt 16.27 min), 381.2053 ([M + H]^+^, Rt 16.38 min), 191.1069 ([M + H]^+^, Rt 16.53 min), 191.1065 ([M + H]^+^, Rt 16.65 min), 192.1106 ([M + H]^+^, Rt 16.76 min), 381.2056 ([M + H]^+^, Rt 16.76 min) and 382.2109 ([M + H]^+^, Rt 16.76 min) are far from the centre of the loadings plot suggesting that these components were highly varied in these 27 samples during whole growth period.

### Quantitative analysis of compounds in *Angelica sinensis*

Quantification was performed using linear calibration plots of peak areas and concentration. The proposed UPLC-MS/MS method was subsequently applied to determine four chemical markers including Z-ligustilide (**7**), butylphthalide (**10**), Z-butylidenephalide (**11**), linoleic acid (**12**) in *Angelica sinensis*.

**Figure 8:**
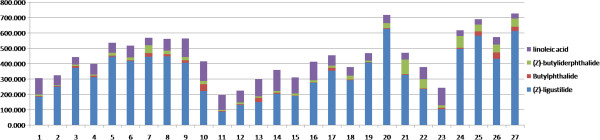
**The contents (ng/g) of 4 investigated compounds in *Angelica sinensis *during growth.**

The results (Figure 8) showed there were remarkable differences in their contents during growth. These four chemical markers’ concentrations were relatively higher in September and October. The result was in accordance with traditional harvesting period.

## Experimental

### Materials and reagents

Acetonitrile (HPLC grade) was purchased from TEDIA Company Inc. (Fairfield, USA); formic acid was obtained from Merck KGaA (Darmstadt, Germany); Ultra-pure water was purified by an EPED super purification system (Nanjing, China). The distilled water was used for the extraction and preparation of samples. Ferulic acid, (Z)-ligustilide and levistolide A were purchased from Chengdu must bio-technology Co., Ltd. Butylphthalide, (Z)-butyliderphthalide were purchased from Sichuan Xianxin Biotech Co., Ltd. Linoleic acid was obtained from Sigma-Aldrich (St. Louis, MO, USA)*,* roots of *Angelica sinensis* at different stages of growth were harvested from Ma Zichuan Village, Min County, Gansu Province, China. Table 2 shows the detail information of these 27 samples. All other chemicals used were of analytical grade.

**Table 2:** **Sample information of *Angelica sinensis *in different growth periods**

**Sample ** **No.**	**Collecting time**		**Cluster in PCA score plot**	**Sample No.**	**Collecting time**		**Cluster in PCA score plot**
1	August 30^th^	1^st^ year	I	15	September 25^th^	2^nd^ year	I
2	September 13^th^	1^st^ year	I	16	October 5^th^	2^nd^ year	I
3	October 1^st^	1^st^ year	I	17	October 14^th^	2^nd^ year	II
4	October 8^th^	1^st^ year	I	18	October 24^th^	2^nd^ year	II
5	March 29^th^	2^nd^ year	II	19	November 7^th^	2^nd^ year	II
6	April 4^th^	2^nd^ year	II	20	April 30^th^	3^rd^ year	III
7	May 29^th^	2^nd^ year	III	21	May 29^th^	3^rd^ year	III
8	June 15^th^	2^nd^ year	III	22	June 28^th^	3^rd^ year	III
9	June 28^th^	2^nd^ year	III	23	July 26^th^	3^rd^ year	IV
10	July 12^th^	2^nd^ year	IV	24	August 30^th^	3^rd^ year	III
11	July 26^th^	2^nd^ year	IV	25	September 13^th^	3^rd^ year	III
12	August 15^th^	2^nd^ year	I	26	September 30^th^	3^rd^ year	III
13	August 27^th^	2^nd^ year	I	27	October 29^th^	3^rd^ year	III
14	September 14^th^	2^nd^ year	I				

### Sample preparation

For each sample, an accurately weighed 0.5 g of the dried powder was introduced into a 50 mL calibrated flask and 20 mL of 70% methanol were added. The weight of this flask (with sample and methanol) was recorded. The calibrated flask was covered and soaked for one hour at room temperature. Then the powder was extracted in an ultrasonic cleaner for 45 min. And 70% methanol was used to make up the change of weight. All extracted samples were centrifuged at 3000 rpm for 5 min and were passed through a 0.22 μm syringe filter before they were injected for UPLC/TOFMS analysis. Sample No.14 was chosen to inject 5 times for precision and accuracy analysis. The similarity of precision was higher than 0.98.

### Standard preparation

All 6 reference standards were dissolved by methanol to final concentrations of 0.27 mg · mL^-1^ for ferulic acid; 0.24 mg · mL^-1^ for levistolide A; 0.22 mg · mL^-1^ for (Z)-ligustilide; 0.20 mg · mL^-1^ for butylphthalide; 0.20 mg · mL^-1^ for (Z)-butyliderphthalide; 0.26 mg · mL^-1^ for linoleic acid.

### Liquid chromatography

The UPLC-ESI-MS system was performed on a Waters Acquity UPLC system (Waters Corp., Milford, MA, USA), equipped with a binary solvent delivery system, a conditioned autosampler at 4°C and a photo-diode array detector (PDA) system. Chromatographic separation was carried out on an ACQUITY UPLC™ BEH C_18_ column (100 mm × 2.1 mm I.D., 1.7 μm particle size) (Waters, Milford, USA). The column was maintained at 30°C. The mobile phase was gradient elution mixed with solvents A (0.1% aqueous formic acid, V/V) and B (acetonitrile): 0 min 97% A, 8 min 57% A, 12 min 57% A, 16 min 30% A, 17 min 30% A, 20 min 3% A, 22 min 97% A, with the flow rate of 0.4 mL/min.

### MS

The MS analysis was performed on a Waters ACQUITY™ Synapt Q-TOF mass spectrometer connected to the Waters Acquity UPLC system via an electrospray ionization interface (ESI). ESI mass spectra were acquired in both positive (ESI^+^) and negative (ESI^-^) electrospray ionization modes by scanning over the m/z range 100–1000. The optimized conditions were as follows: (1) ESI^+^ mode, capillary voltage 3 kV; sample cone voltage 30 V; extraction cone 2.0 V; source temperature 120°C; desolvation temperature 350°C; cone gas flow 50 L/h; desolvation gas flow 600 L/h, and (2) ESI^-^ mode, similar conditions as ESI^+^ mode.

Mass spectrometry detection for quantification was performed using a Xevo Triple Quadrupole MS (Waters Corp., Milford, MA, USA) equipped with an electrospray ionization source (ESI). The ESI-MS spectra were acquired in positive ion multiple reaction monitoring (MRM) mode. The detailed ion transition data for MRM mode was shown in Table 3.

**Table 3:** Related MS data of the 4 target markers detected in MRM mode

** Analytes**	**t**_**R **_**(min)**	**Quantitative ion (m/z)**
Butylphthalide	10.47	145
Z-Ligustilide	12.50	91
Z-Butylidenephalide	12.83	127
Linoleic acid	19.74	279

### Accurate mass measurement

Data were centroided during acquisition using independent reference lock-mass ions via the Lockspray™ interface to ensure mass accuracy and reproducibility. The [M − Glucose] ^−^ and [M-Rutinose]^+^ ions of Rutin at m/z 300.0270 and m/z 303.0505 were used as the lock mass in negative and positive electrospray ionization mode, respectively. Rutin was used as the reference compound at a concentration of 0.001 μg/μL and the infusion flow rate of 0.26 mL/min to ensure mass accuracy and reproducibility. During metabolite profiling experiments, centroided data were acquired for each sample from 100 to 1000 Da with a 0.20 s scan time and a 0.02 s inter scan delay over a 23 min analysis time. This produced an average of 13 scans across a peak of average width 0.06 min obtained using UPLC.

### Chemometric data analysis

The UPLC-QTOF/MS data of 27 samples were analyzed to identify potential discriminate variables. The peak finding, peak alignment, and peak filtering of ESI^+^ raw data were carried out with MarkerLynx applications manager version 4.1 (Waters). The parameters used were Rt range 0–23 min, mass range 100–1000 Da, mass tolerance 0.05 Da, internal standard detection parameters were deselected for peak retention time alignment, isotopic peaks were excluded for analysis, and noise elimination level was set at 6.00. The UPLC-PDA-MS chromatographic fingerprints of 27 *Angelica sinensis* samples were generated by Matlab 6.5 software.

## Conclusions

In this study UPLC/TOFMS had been demonstrated to be a powerful tool for metabolite profiling of *Angelica sinensis* during growth and it is applicable for analysis and evaluation of complex herbal medicines. The proposed analytical method coupled with chemometrics data analysis technique is used as a powerful tool to differentiate phytochemical compositions among samples from *Angelica sinensis* at different stages of growth for quality control. Results from this study indicated that accumulation of secondary metabolites in *Angelica sinensis* is closely related to the growth periods. To study the alterations of secondary metabolites of *Angelica sinensis* during growth can provide evidence for choosing the suitable harvest time.

## Competing interest

The authors declare that they have no competing interests.

## Authors’ contributions

YLW planed and supervised the whole work; YYQ carried out the experiments and drafted the manuscript. RNS, HY, XBP, YWY and YJS participated in experiments. All authors read and approved the final manuscript.

## References

[B1] China Pharmacopoeia Committee Pharmacopoeia of the People’s Republic of China 2010 Beijing, China: China Chemical Industry Press 124

[B2] Wagner H Bauer R Xiao PG Chinese Drug Monographs and Analysis Angelica sinensis 2001 3 1 2

[B3] Yi LZ Liang YZ Wu H Yuan DL The analysis of Radix Angelicae Sinensis (Danggui) J Chromatogr A 2009 1216 1991 2001 10.1016/j.chroma.2008.07.033 18667208

[B4] Fiehn O Metabolomics - the link between genotypes and phenotypes Plant Mol Biol 2002 48 155 171 10.1023/A:1013713905833 11860207

[B5] Verouden MPH Westerhuis JA van der Werf MJ Smilde AK Exploring the analysis of structured metabolomics data Chemometrics and Intelligent Laboratory Systems 2009 98 88 96 10.1016/j.chemolab.2009.05.004

[B6] Castillo S Gopalacharyulu P Yetukuri L Orešič M Algorithms and tools for the preprocessing of LC–MS metabolomics data Chemometrics and Intelligent Laboratory Systems 2011 108 23 32 10.1016/j.chemolab.2011.03.010

[B7] Ioset KN Nyberg NT Van Diermen D Malnoe P Hostettmann K Shikov AN Jaroszewski JW Metabolic profiling of Rhodiola rosea rhizomes by ^1^H NMR spectroscopy Phytochem Anal 2011 22 158 165 10.1002/pca.1262 20848394

[B8] Gad HA El-Ahmady SH Abou-Shoer MI Al-Azizi MM Application of Chemometrics in Authentication of Herbal Medicines: A Review Phytochem Anal 2012 n/a n/a 10.1002/pca.2378 22678654

[B9] Xue SY Li ZY Zhi HJ Sun HF Zhang LZ Guo XQ Qin XM Metabolic fingerprinting investigation of Tussilago farfara L. by GC–MS and multivariate data analysis Biochem Syst Ecol 2012 41 6 12

[B10] Zhi HJ Qin XM Sun HF Zhang LZ Guo XQ Li ZY Metabolic Fingerprinting of Tussilago farfara L. Using ^1^H-NMR Spectroscopy and Multivariate Data Analysis Phytochem Anal 2012 23 492 501 10.1002/pca.2346 22371211

[B11] Jansen JJ Smit S Hoefsloot HCJ Smilde AK The photographer and the greenhouse: how to analyse plant metabolomics data Phytochem Anal 2010 21 48 60 10.1002/pca.1181 19904732

[B12] Tianniam S Tarachiwin L Bamba T Kobayashi A Fukusaki E Metabolic profiling of Angelica acutiloba roots utilizing gas chromatography–time-of-flight–mass spectrometry for quality assessment based on cultivation area and cultivar via multivariate pattern recognition J Biosci Bioeng 2008 105 655 659 10.1263/jbb.105.655 18640606

[B13] Xiang Z Wang XQ Cai XJ Zeng S Metabolomics Study on Quality Control and Discrimination of Three Curcuma Species based on Gas Chromatograph-Mass Spectrometry Phytochem Anal 2011 22 411 418 10.1002/pca.1296 21433157

[B14] Ma C Wang H Lu X Xu G Liu B Metabolic fingerprinting investigation of Artemisia annua L. in different stages of development by gas chromatography and gas chromatography–mass spectrometry J Chromatogr A 2008 1186 412 419 10.1016/j.chroma.2007.09.023 17915234

[B15] Yi LZ Yuan DL Liang YZ Xie PS Zhao Y Fingerprinting alterations of secondary metabolites of tangerine peels during growth by HPLC–DAD and chemometric methods Anal Chim Acta 2009 649 43 51 10.1016/j.aca.2009.07.009 19664461

[B16] Allwood JW Goodacre R An introduction to liquid chromatographyâ mass spectrometry instrumentation applied in plant metabolomic analyses Phytochem Anal 2010 21 33 47 10.1002/pca.1187 19927296

[B17] De Vos RCH Moco S Lommen A Keurentjes JJB Bino RJ Hall RD Untargeted large-scale plant metabolomics using liquid chromatography coupled to mass spectrometry Nat Protoc 2007 2 778 791 10.1038/nprot.2007.95 17446877

[B18] Chan ECY Yap SL Lau AJ Leow PC Toh DF Koh HL Ultra-performance liquid chromatography/time-of-flight mass spectrometry based metabolomics of raw and steamed Panax notoginseng Rapid Commun Mass Spectrom 2007 21 519 528 10.1002/rcm.2864 17238214

[B19] Dan M Su M Gao X Zhao T Zhao A Xie G Qiu Y Zhou M Liu Z Jia W Metabolite profiling of Panax notoginseng using UPLC–ESI-MS Phytochemistry 2008 69 2237 2244 10.1016/j.phytochem.2008.04.015 18550132

[B20] Li SL Song JZ Qiao CF Zhou Y Qian K Lee KH Xu HX A novel strategy to rapidly explore potential chemical markers for the discrimination between raw and processed Radix Rehmanniae by UHPLC-TOFMS with multivariate statistical analysis J Pharm Biomed Anal 2010 51 812 823 10.1016/j.jpba.2009.10.002 19879709

[B21] Lin LZ He XG Lian LZ Wayne K Jerry E Liquid chromatographic–electrospray mass spectrometric study of the phthalides of *Angelica sinensis* and chemical changes of Z-ligustilide J Chromatogr A 1998 810 71 79 10.1016/S0021-9673(98)00201-5

[B22] Wang YL Liang YZ Chen BM High-performance liquid chromatography with atmospheric pressure chemical ionization and electrospray ionization mass spectrometry for analysis of Angelica sinensis Phytochem Anal 2007 18 265 274 10.1002/pca.968 17623360

[B23] Lu GH Chan K Liang YZ Leung K Chan CL Jiang ZH Zhao ZZ Development of high-performance liquid chromatographic fingerprints for distinguishing Chinese Angelica from related umbelliferae herbs J Chromatogr A 2005 1073 383 392 10.1016/j.chroma.2004.11.080 15909545

[B24] Liu HM Liu XZ Liu XR Wang XZ Effects of cultivation methods on dry matter accumulating and growth dynamics of Angelica sinensis Chin Tradit Herbal Drugs 2007 38 257 261

[B25] Sun HM Zhang BG Investigation on the Growth Activities of Angelica sinensis(oliv.) Diels. in Gansu Region Chin Agric Sci Bull 2010 26 386 389

[B26] Xu JZ Qi FP Jing YM Liu MR Zhao R Li QP Cai RQ Studies on the Dynamic of Angelicae sinensis in Medicine Formation Period Chin Med Mat 1997 20 325 327

